# Eutocic delivery vs. cesarean section: a multicenter retrospective study of factors influencing cesarean rates in Robson groups 1, 2a, 3, and 4a across birth centers

**DOI:** 10.3389/fmed.2025.1635018

**Published:** 2025-10-24

**Authors:** Alessandra Musicco, Gianfranco Sfregola, Vincenzo Dicuonzo, Tatiana Bolgeo, Virginia Milone, Grazia Dicuonzo, Paolo Iovino, Federico Ruta

**Affiliations:** ^1^OPI BAT, Trani, Italy; ^2^ASL BT, Barletta, Italy; ^3^ANT Foundation, Trani, Italy; ^4^Azienda Ospedaliero Universitario Alessandria – SS Antonio e Belgio e C. Arrigo, Torino, Italy; ^5^Department of Economics, Management and Business Law, University of Bari Aldo Moro, Bari, Italy; ^6^Health Sciences Department, University of Florence, Florence, Italy; ^7^Azienda Sanitaria Locale Barletta Andria Trani, Andria, Italy

**Keywords:** cesarean section, Robson classification, obstetric care, labor induction, maternal health, delivery mode, health policies, gender medicine

## Abstract

**Objective:**

Cesarean deliveries are increasing rapidly worldwide. Although their primary indication is when vaginal delivery is not feasible, there appears to be an overutilization of this procedure, even in the absence of clear medical or obstetric indications. This exposes women to short-term and long-term adverse outcomes. This study aimed to investigate factors associated with cesarean section (CS) in nulliparous and multiparous women undergoing spontaneous and induced labor, focusing on Robson groups 1, 2a, 3, and 4a in birth centers of the Apulia Region (Italy) in 2019.

**Methods:**

This multicenter retrospective observational study used data from the Delivery Room Registers of 14 facilities in the Apulia Region in 2019, covering 14,331 women. Inclusion criteria were belonging to Robson groups 1, 2a, 3, or 4a. Exclusion criteria were stillbirths and deliveries occurring in ambulances or at home. The final sample consisted of 9,992 women. Multilevel binary logistic regression models were performed to assess the impact of Robson groups and their combinations on the likelihood of CS. Chi-squared and Fisher’s exact tests were used to examine the distribution of CS across facilities.

**Results:**

Among women with spontaneous labor, multiparity was protective against CS compared with nulliparity (OR = 0.44, *p* < 0.001). Similarly, in induced labor, multiparity remained protective (OR = 0.46, *p* < 0.001). Women undergoing induction were approximately four times more likely to deliver via CS compared with those in spontaneous labor (OR = 3.87, *p* < 0.001). Overall, multiparous women were substantially less likely to undergo CS compared with nulliparous women (OR = 0.18, *p* < 0.001). Significant variability in CS rates across facilities was observed for all Robson groups (*p* < 0.001).

**Conclusion:**

Nulliparity and induction of labor were strongly associated with increased risk of CS. These factors should be carefully considered in clinical decision-making to help reduce unnecessary CS and mitigate associated adverse health outcomes.

## 1 Introduction

Caesarean section (CS) is a potentially life-saving procedure. However, ensuring timely and safe access to CS remains a major challenge for health systems in countries with high maternal mortality (1). Conversely, in many other settings, women are increasingly undergoing CS without clear, evidence-based indications, contributing to the worldwide secular trend of rising CS rates (2, 3). When performed for medical reasons, CS can prevent severe morbidity and mortality for both mother and infant (4, 5). However, this procedure is also associated with significant short- and long-term risks for the mother–child dyad, underscoring the importance of limiting its use to situations of genuine medical necessity. Reported risks include surgical complications (e.g., inflammatory and neuroendocrine response, organ injury, and fluid and heat loss), procedure-related adverse outcomes (e.g., abdominal pain, bladder or ureteral damage, hysterectomy, and thromboembolic disease), anesthesia-related complications (linked to drugs, techniques, or invasive monitoring), and broader biological effects of childbirth.

Cost-effectiveness is also a crucial consideration when evaluating the use of CS. Across different healthcare systems, vaginal birth has consistently been shown to be less costly than CS, largely due to shorter hospital stays, fewer postoperative complications, and reduced use of medical resources (5). For example, a cost-effectiveness analysis from Colombia demonstrated that spontaneous vaginal delivery was both less expensive and more effective than elective CS for low-risk women, reinforcing the view that vaginal birth is economically preferable and carries important implications for public health policy in middle-income countries (6). Similarly, in Brazil’s private healthcare system, vaginal birth was shown to be less costly and equally or more effective, even in a context where cesarean delivery is often culturally and institutionally favored (7).

Over the past two decades, increasing debate has surrounded the appropriateness of performing CS at maternal request or on the advice of healthcare professionals in the absence of clear medical indications. Issues of safety, cost, women’s rights and preferences, along with both maternal and professional satisfaction, have all been central to this discussion, highlighting the interplay between medical, ethical, and individual considerations (8), which recent literature has further expanded with strategies and ethical advances (9–11).

In recent years, several systematic reviews and institutional statements have emphasized strategies to reduce unnecessary CS, including audit and feedback programs, standardized labor management protocols, and quality-improvement initiatives aimed at primary cesarean births (9, 10). At the same time, multilevel analyses have highlighted how health system drivers and organizational practices contribute to the variability of CS rates (11). Ethical aspects are also increasingly relevant, with women’s autonomy, informed choice, and the balance between maternal preferences and clinical appropriateness gaining prominence in contemporary obstetric care.

The rising global prevalence of CS remains a major public health concern (6, 7). In a setting where CS is available, optimal rates are expected. Recent studies suggest that the ideal CS rate lies between 10 and 20% of all births (12, 13). Nevertheless, despite the introduction of multiple protocols and guidelines for intrapartum care and the publication of official reports comparing CS rates across hospitals, substantial variability persists. This variability has been attributed to inconsistent application of guidelines, lack of uniform regulation regarding maternal requests, defensive medical practices, uneven adherence to standards, and increased surgical activity linked to remuneration mechanisms within the National Health Care System.

Therefore, the purpose of this study was to investigate CS trends in 2019, identifying maternal characteristics associated with a higher risk of undergoing CS, while accounting for institutional and obstetric factors. We also estimated the difference in CS prevalence within the same Robson groups across different inpatient facilities in the Apulia region (Italy). The findings are intended to inform the development of public health benchmarks for maternal and neonatal health programs, guide the design of innovative policies and clinical guidelines, and support risk factor screening and awareness campaigns for pregnant women.

## 2 Methods

### 2.1 Design

This was a multicentric retrospective observational study.

### 2.2 Instruments and data collection

Data collection followed three steps. First, we obtained formal authorization from the Health Management of each participating facility. Next, data were retrieved from 14 facilities in the Apulia Region (Italy). Data were collected in digital format (Microsoft Excel, Microsoft 365) from the Delivery Room Registers (parallel registry and supporting care activity), including Robson classification variables and information on compliance with operational standards. All women meeting the inclusion criteria were considered eligible and included in the analysis; no data extraction or sampling was performed. Subsequently, data were analyzed using descriptive statistics and inferential procedures, including multilevel logistic regression models, chi-squared test, and Fisher’s exact test. The Robson classification, proposed by the World Health Organization in 2015, is a global standard for assessing, monitoring, and comparing CS rates. It categorizes women into 10 mutually exclusive and totally inclusive groups based on obstetric characteristics (14). It has been widely validated, demonstrating high validity (accurately reflecting obstetric risks), reliability (consistent application across settings), and reproducibility (stable results over time). Its high interrater agreement and responsiveness make it an effective tool for monitoring trends and informing both clinical practice and policy decisions.

### 2.3 Ethical considerations

Patients were not involved in developing the research questions, study design, or participant recruitment. Given the retrospective design, informed consent was not required. All data were fully anonymized prior to analysis; no direct identifiers (e.g., name, surname, and date of birth) and records could not be traced back to individual participants. The use of anonymized data ensured full compliance with ethical and legal standards for data protection.

### 2.4 Sample

We collected data on 9,992 women from the Delivery Room Registers of birth centers in the Apulia Region (Italy) in 2019. Inclusion criteria were Robson groups 1 (nulliparous, spontaneous labor), 2a (nulliparous, induced labor), 3 (multiparous, spontaneous labor), and 4a (multiparous, induced labor). We excluded women with stillbirths, those with a gestational age of ≤ 28 weeks (as these cases concern fetal intrauterine death management and specific clinical recommendations), and women who delivered in ambulances or at home. We also excluded women belonging to Robson groups 2b, 4b, and 5–10. This choice was adopted as these groups represent absolute maternal and/or fetal clinical recommendations for CS. These groups were excluded because they represented clinical scenarios with absolute maternal and/or fetal indications for CS established based on the woman’s clinical records prior to delivery.

### 2.5 Statistical analysis

All analyses were performed using STATA^®^ MP 15. Sample characteristics were described as means (SD) for quantitative variables and frequencies (%) for categorical variables. No missing data were present for the variables included in the analysis; therefore, complete case analysis was applied. Because patients were hierarchically nested within 14 healthcare facilities, we applied multilevel binary logistic regression models with a random intercept at the facility level to account for intra-center correlation. This approach adjusts for unobserved heterogeneity across centers while estimating the fixed effects of Robson groups on CS risk. Random slopes were not included, as the objective of the analysis was to assess overall associations between Robson groups and cesarean delivery risk, rather than to model variation in these associations across facilities. The independent variables were the four Robson groups or their combinations, while the dependent variable was dichotomized as 0 = vaginal delivery and 1 = cesarean delivery. We estimated the effects of all four Robson groups individually, group 3 vs. group 1, group 4a vs. group 2a, groups 2a + 4a vs. groups 1 + 3, and groups 3 + 4a vs. groups 1 + 2a. Results are presented as odds ratios (ORs) with 95% confidence intervals (CIs) and *p*-values. To examine differences in CS distribution across facilities, we used Pearson’s chi-squared test (or Fisher’s exact test when expected cell counts were <5). Because the chi-squared test only indicates overall significance, we performed *post-hoc* analyses of adjusted standardized residuals to identify specific facility–Robson group combinations contributing to the association. Adjusted residuals follow an approximate standard normal distribution, with values exceeding ±1.96 indicating cells where the observed frequency significantly deviates from expectation under independence. To account for multiple testing, the Bonferroni correction was applied. With four Robson groups analyzed, the adjusted significance threshold was set at a *p*-value of < 0.013 (0.05/4).

## 3 Results

### 3.1 Sample characteristics

Table 1 presents the characteristics of the participants (*n* = 9,992) and the healthcare settings. Overall, 15.7% of women were delivered by CS, with the largest proportion belonging to Robson group 1 (43.3%). Slightly more than half of the women (52.2%) delivered in hospitals offering pharmacological pain control (analgesia during childbirth). Only 62.7% of the sample had access to a facility with a 24-h operating room available for obstetric emergencies. In addition, approximately 75% of the women delivered in facilities with areas dedicated to the management of physiological/natural labor, and 87.2% were in hospitals where the number of labor-birthing rooms was appropriate to the annual number of births.

**Table 1:** Characteristics of women and admission settings (*n* = 9,992).

Characteristic	*n* (%)
Cesarean delivery	1,568 (15.7)
Robson group	
Group 1	4,325 (43.3)
Group 2a	1,692 (16.9)
Group 3	3,416 (34.2)
Group 4a	559 (5.6)
Childbirth analgesia	5,242 (52.5)
Operating room	6,268 (62.7)
Physiology-dedicated area	7,529 (75.4)
Labor room availability	8,718 (87.2)

Data are presented as *n* (%). Robson groups: group 1: nulliparous, single cephalic, ≥ 37 weeks, in spontaneous labor. Group 2a, nulliparous, single cephalic, ≥ 37 weeks, induced. Group 3: multiparous, single cephalic, ≥ 37 weeks, in spontaneous labor. Group 4a: multiparous, single cephalic, ≥ 37 weeks, induced.

### 3.2 Results of the logistic regression

Table 2 presents the results of the logistic regression comparing different Robson group combinations in relation to cesarean delivery. Compared with group 1 (nulliparous, spontaneous labor), women in group 2a (nulliparous, induced labor) were approximately three times more likely to undergo CS (OR = 3.19, *p* < 0.001). In contrast, women in groups 3 and 4a (multiparous, spontaneous, and induced labor, respectively) were less likely to have CS. Among women with spontaneous labor, multiparous women (group 3) had 56% lower odds of undergoing CS compared with nulliparous women (group 1) (OR = 0.44, *p* < 0.001). Similarly, among those with induced labor, multiparous women (group 4a) had 54% lower odds of CS compared with nulliparous women (group 2a) (OR = 0.46, *p* < 0.001). When considering the full sample, induction of labor was associated with approximately 4-fold higher odds of CS compared with spontaneous labor (OR = 3.87, *p* < 0.001). Overall, multiparous women were significantly less likely to undergo CS than nulliparous women (OR = 0.18, *p* < 0.001).

**Table 2:** Multilevel logistic regression models for cesarean delivery across Robson groups.

Comparison	OR (95% CI)	*p*-value
Group 2a vs. 1	**3.19 (2.76–3.68)**	<0.001
Group 3 vs. 1	**0.19 (0.16–0.23)**	<0.001
Group 4a vs. 1	**0.70 (0.53–0.92)**	0.013
Group 3 vs. 1 (contrast)	**0.44 (0.40–0.48)**	<0.001
Group 4a vs. 2a (contrast)	**0.46 (0.40–0.54)**	<0.001
Groups 2a + 4a vs. 1 + 3	**3.87 (3.40–4.40)**	<0.001
Groups 3 + 4a vs. 1 + 2a	**0.18 (0.15–0.21)**	<0.001

OR, odds ratio; CI, confidence interval; *p*, *p*-value. Estimates are from multilevel logistic regression models with patients nested within hospitals (random intercept). Statistically significant associations (*p* < 0.05) are shown in bold.

Robson groups are defined as follows: group 1: nulliparous, single cephalic, ≥ 37 weeks, in spontaneous labor. Group 2a, nulliparous, single cephalic, ≥ 37 weeks, induced. Group 3: multiparous, single cephalic, ≥ 37 weeks, in spontaneous labor. Group 4a: multiparous, single cephalic, ≥ 37 weeks, induced.

### 3.3 Proportion of cesarean delivery stratified by Robson group and hospital facility

Table 3 reports the contingency table of CS rates across Robson groups and hospital facilities. For each group, a significant association was observed between facility and type of delivery (all *p* < 0.001). Specifically, the frequency of CS among women in group 2a was significantly higher than expected in seven hospitals, followed by group 1, where higher-than-expected frequencies were observed in four hospitals. Notably, San Paolo Hospital in Bari showed higher-than-expected CS rates across all Robson groups.

**Table 3:** Cesarean delivery rates with 95% confidence intervals across Robson groups and hospitals in the Apulia Region (Italy).

Hospital		Group 1 (*n* = 4,325)		Group 2a (*n* = 1,692)		Group 3 (*n* = 3,416)		Group 4a (*n* = 559)
	*n*	CD *n* (%, 95Cis)	*n*	CD *n* (%, 95Cis)	*n*	CD *n* (%, 95Cis)	*n*	CD *n* (%, 95Cis)
Mons. Dimiccoli, Barletta	281	25 (8.9%, 6.1–12.8)	75	21 (28.0%, 19.1–39.0)	273	10 (3.7%, 2.0–6.6)	20	0 (0.0%, 0.0–16.1)
V. Emanuele II, Bisceglie	146	20 (13.7%, 9.0–20.2)	76	24 (31.6%, 22.2–42.7)	141	2 (1.4%, 0.4–5.0)	25	3 (12.0%, 4.2–30.0)
L. Bonomo, Andria	201	21 (10.4%, 6.9–15.4)	122	**58 (47.5%, 38.9–56.3)**	199	0 (0.0%, 0.0–1.9)	40	3 (7.5%, 2.6–19.9)
D. Camberlingo, F. Fontana	200	44 (22.0%, 16.8–28.2)	100	**43 (43.0%, 33.7–52.8)**	137	1 (0.7%, 0.1–4.0)	27	4 (14.8%, 5.9–32.5)
Di Summa—Perrino, Brindisi	432	**170 (39.4%, 34.9–44.0)**	37	**29 (78.4%, 62.8–88.6)**	225	**21 (9.3%, 6.2–13.8)**	18	4 (22.2%, 9.0–45.2)
Umberto I, Corato	209	49 (23.4%, 18.2–29.6)	79	**36 (45.6%, 34.5–57.2)**	201	5 (2.5%, 1.1–5.7)	40	3 (7.5%, 2.6–19.9)
San Giacomo, Monopoli	292	37 (12.7%, 9.3–17.0)	20	**20 (100%, 83.9–100)**	232	5 (2.2%, 0.9–5.0)	3	**3 (100%, 43.9–100)**
R. Miulli, Acquaviva delle Fonti	319	27 (8.5%, 5.9–12.0)	436	110 (26.6%, 22.6–31.0)	373	61 (16.4%, 13.0–20.5)	132	11 (8.3%, 4.7–14.3)
Mater Dei Hospital, Bari	288	**74 (25.7%, 21.0–31.0)**	78	**43 (55.1%, 43.8–66.0)**	192	12 (6.3%, 3.6–10.8)	32	5 (15.6%, 6.8–31.8)
San Paolo, Bari	194	**62 (32.0%, 25.8–38.8)**	45	**26 (57.8%, 43.2–71.2)**	173	**14 (8.1%, 4.9–13.1)**	25	**9 (36.0%, 19.9–56.5)**
Di Venere, Bari Carbonara	608	93 (15.3%, 12.7–18.4)	259	68 (26.3%, 21.3–32.0)	347	17 (4.9%, 3.1–7.8)	43	1 (2.3%, 0.4–12.0)
Occidentale, Castellaneta	154	27 (17.5%, 12.3–24.3)	41	9 (22.0%, 12.0–36.8)	132	8 (6.1%, 3.1–11.5)	21	3 (14.3%, 4.9–35.1)
S.S. Annunziata, Taranto	494	31 (6.3%, 4.5–8.9)	256	38 (14.8%, 11.0–19.7)	401	6 (1.5%, 0.7–3.3)	88	7 (8.0%, 3.9–15.7)
Teresa M. Mascia, San Severo	204	43 (21.1%, 16.0–27.2)	44	16 (36.4%, 23.4–51.7)	236	**16 (6.8%, 4.2–10.8)**	38	6 (15.8%, 7.4–30.5)
G. Tatarella, Cerignola	303	**84 (27.7%, 22.9–33.1)**	24	12 (50.0%, 31.9–68.1)	154	15 (9.7%, 5.9–15.6)	7	2 (28.6%, 8.2–64.1)
Pearson chi-squared test (*p*-value), Cramer’s V	287.95 (<0.001), 0.069		184.67 (<0.001), 0.088		76.15 (<0.001), 0.040		39.51* (<0.001), 0.071	

CD, cesarean delivery; CI, confidence interval. Proportions are reported as *n* (% with 95% CI, Wilson method).

Chi-squared tests assess the association between hospital facility and cesarean delivery within each Robson group. Cramér’s V is reported as a measure of effect size (0.1 = small, 0.3 = medium, and 0.5 = large). *In group 4a, 14 cells (46.7%) had expected counts less than 5; therefore, Fisher’s exact test was performed. *Fisher’s exact test was performed with a Monte Carlo simulation based on 10,000 sampled tables. Absolute percentages and numbers in bold indicate values whose expected residuals are greater than 1.96 (i.e., values statistically greater than expected if the two variables were independent). Robson groups are defined as follows: group 1: nulliparous, single cephalic, ≥ 37 weeks, in spontaneous labor. Group 2a, nulliparous, single cephalic, ≥ 37 weeks, induced. Group 3: multiparous, single cephalic, ≥ 37 weeks, in spontaneous labor. Group 4a: multiparous, single cephalic, ≥ 37 weeks, induced.

## 4 Discussion

### 4.1 Interpretation of findings and clinical implications

This study aimed to investigate delivery patterns in the Apulian Region (Italy), with a particular focus on the prevalence of CS vs. vaginal delivery. Women were analyzed according to their Robson group classification, and the results presented in Tables 2, 3 provide important insights into the factors influencing CS across groups and facilities.

A key finding of this study is the significant difference in CS rates between nulliparous and multiparous women in both spontaneous and induced labor. Specifically, women who had not previously given birth and underwent induction were approximately three times more likely to deliver with CS than those in spontaneous labor. These findings are consistent with previous studies demonstrating that nulliparity and induction of labor are major risk factors for CS. We also found that multiparous women, whether in spontaneous or induced labor, were less likely to undergo CS than their nulliparous counterparts. The protective effect of multiparity on CS has been well documented in previous studies.

In the overall sample, women with induced labor were approximately four times more likely to deliver by CS compared with those in spontaneous labor. This strong association highlights the clinical challenges linked to induction. Our findings are consistent with those of Caughey et al. (15), who, in a study of more than 20,000 women, reported that labor induction was associated with a 2.5-fold higher risk of CS compared with spontaneous labor.

The variation in CS rates across healthcare facilities in the Apulia Region underscores the complex interplay of factors influencing delivery outcomes. Beyond clinical and obstetric factors, the variability in cesarean section rates between hospitals may reflect differences in organizational culture, availability of resources, and professional attitudes toward labor management. Non-clinical determinants, such as physician preferences, institutional protocols, medico-legal pressures, and patient expectations, have been shown to significantly influence the likelihood of cesarean delivery. These considerations provide a broader interpretive framework, supported by the literature, while our empirical findings remain limited to the observed variability across Robson groups and facilities.

The discussion of these results should therefore extend beyond descriptive comparisons. Alternative hypotheses must be considered, including how institutional protocols, professional decision-making styles, and the expectations generated by community norms for childbirth may shape cesarean section rates. Such differences illustrate that variability across facilities is not merely the result of medical indications but also of organizational and cultural dynamics. Addressing this complexity requires the harmonization of clinical pathways, implementation of evidence-based guidelines, and strategies that promote shared decision-making with women and families.

Recent evidence further supports this interpretation. Gupta et al. demonstrated that maternal and institutional factors significantly influence induction success (16). Selin et al. also highlighted population-level predictors of successful induction (17). More recently, Karlsson et al. found that induction of labor was associated with an increased risk of cesarean delivery in nulliparous women (18).

Notable disparities were observed, such as unexpectedly high CS rates in group 2a across seven hospital settings, and in group 1 across four hospitals. These findings suggest that hospital policies, resource allocation, and clinical practices can strongly affect the prevalence of CS, highlighting the need for more individualized and context-sensitive approaches to obstetric care (13, 19, 20).

Research by Betrán et al. (13) highlights the global rise in cesarean delivery rates, with evidence indicating that hospital policies, clinical practices, and resource allocation play a major role in these trends. Practices such as the routine use of CS in certain clinical scenarios and the influence of healthcare infrastructure can contribute to regional variability. Similarly, Vogel et al. (21) emphasize the value of the Robson classification in identifying differences in CS rates across different settings. Within this framework, the classification is particularly useful for comparing local patterns, such as those observed in Apulian hospitals, where institutional and organizational factors may explain part of the heterogeneity in CS prevalence.

Our results suggest that CSs were performed more frequently than expected in our sample, as many women presented with favorable clinical conditions for vaginal delivery. This observation was verified by reviewing clinical records and the data used for the Robson classification, which provided detailed obstetric profiles. These findings are consistent with previous research by Gibbons et al. (19) and Vogel et al. (21), both of which support our observations regarding group 2a. Moreover, Vogel et al. (21) highlighted the influence of hospital-level factors, such as staffing patterns and clinical protocols, on shaping variations in CS rates across facilities. According to the literature, non-clinical factors often play a decisive role in choosing the delivery mode. However, our findings indicate that nulliparity and induction of labor are strong predictors of CS. Finally, the regional and intra-regional variability observed in Apulia appears to reflect differences in care processes affecting women in Robson groups 1, 2a, 3, and 4a.

The implications of our findings extend to both clinical practice and health policy. Effective strategies should aim to align clinical guidelines with institutional capacities to optimize maternal outcomes and reduce unnecessary surgical interventions. Reducing disparities in CS rates requires comprehensive approaches that balance clinical requirements with the principles of patient-centered care (13, 19, 20, 22, 23).

Taken together, these results suggest that differences between facilities may partly be explained by non-clinical drivers, underscoring the importance of considering institutional and cultural contexts when interpreting cesarean section rates (24–27).

The strength of this study is that we collected data from 14 totally different structures of the Apulia Region (Italy), including a relatively large sample, which enhances the generalizability of our findings and reflects real-world practices and outcomes within the regional healthcare system. However, several limitations should be acknowledged. First, although data were extracted from official delivery room registers, there remains the possibility of transcription errors, incomplete reporting, and omissions in diagnostic or procedural coding. These issues are inherent to the use of administrative or registry-based datasets and may have introduced misclassification bias. While quality checks were performed, we cannot fully exclude inaccuracies that could have affected the categorization of Robson groups or the documentation of delivery outcomes. Second, due to the retrospective design and reliance on registry data, we were unable to control for several maternal and neonatal confounders known to influence the likelihood of CS. These include maternal age, pre-pregnancy body mass index, parity history beyond the Robson classification, obstetric comorbidities such as gestational diabetes and hypertension, fetal weight, and fetal presentation. The absence of these variables may have resulted in residual confounding and limited the precision of our effect estimates. Third, we were unable to differentiate between elective and emergency CS, nor to categorize specific indications (e.g., fetal distress, failed induction, or cephalopelvic disproportion). The delivery room register did not include standardized documentation of indications, which limited our ability to directly assess the appropriateness of CS. This represents a key area for improvement in data collection systems, as distinguishing between medical necessity and non-clinical drivers is essential for evaluating adherence to guidelines and informing health policy (28–32).

Future prospective studies should aim to incorporate these variables and ideally link registry data with clinical records to enable more precise categorization of indications and provide a more comprehensive adjustment for both clinical and non-clinical determinants of cesarean delivery (34).

### 4.2 Conclusion and implications for healthcare policies

The increase in cesarean deliveries reported in the scientific literature is largely influenced by factors beyond clinical guidelines (35, 36). CS is often performed in situations where it may be deemed inappropriate, with appropriateness understood as a dimension of quality of care that encompasses technical and scientific validity, acceptability, and relevance to the individual, specific circumstances, and contexts, in line with current knowledge (37–39). For women in Robson groups 1, 2a, 3, and 4a, the mode of delivery appears to be strongly structure-dependent, as shown by the statistical association between facilities and delivery type. Our findings demonstrate the real possibility of achieving CS rates consistent with those recommended by the World Health Organization (23, 35, 36). Health policies should therefore promote education initiatives targeting both the general population and healthcare professionals, focusing on the dissemination and implementation of evidence-based practices in public and private sectors (37–39).

Based on our findings, several implications for policy and practice can be drawn. At the regional level, systematic use of the Robson classification could support benchmarking and continuous monitoring of CS rates (34–36). At the hospital level, harmonization of intrapartum care protocols and regular audit and feedback may help reduce unwarranted variability (24–27, 33). At the clinical level, promoting evidence-based practices such as VBAC, standardized counseling, and shared decision-making could enhance the appropriateness of delivery mode (22, 23, 35). Future studies are needed to design and validate coordinated interventions aimed at minimizing the influence of non-clinical and structure-dependent factors in determining the mode of delivery (37–39).

## Data Availability

The raw data supporting the conclusions of this article will be made available by the authors, without undue reservation.

## References

[1] Ronsmans C Holtz S Stanton C. Socioeconomic differentials in caesarean rates in developing countries: a retrospective analysis. Lancet [Internet]. (2006);368:1516–1523. Available online at: https://linkinghub.elsevier.com/retrieve/pii/S0140673606696396 17071285 10.1016/S0140-6736(06)69639-6

[2] Stjernholm YV Petersson K Eneroth E. Changed indications for cesarean sections. Acta Obstet Gynecol Scand [Internet]. (2010) 89:49–53. doi: 10.3109/00016340903418777 19916892

[3] Betrán AP Merialdi M Lauer JA Bing-Shun W Thomas J Van Look P. Rates of caesarean section: analysis of global, regional and national estimates. Paediatr Perinat Epidemiol [Internet]. (2007) 21:98–113. doi: 10.1111/j.1365-3016.2007.00786.x 17302638

[4] Ye J Betrán AP Guerrero Vela M Souza JP Zhang J. Searching for the optimal rate of medically necessary cesarean delivery. Birth [Internet]. (2014) 41:237–44. doi: 10.1111/birt.12104 24720614

[5] Chen I Opiyo N Tavender E Mortazhejri S Rader T Petkovic J. Non-clinical interventions for reducing unnecessary caesarean section. Cochrane Database Syst Rev [Internet]. (2018) 9:CD005528. doi: 10.1002/14651858.CD005528.pub3 30264405 PMC6513634

[6] Boerma T Ronsmans C Melesse DY Barros AJD Barros FC Juan L Global epidemiology of use of and disparities in caesarean sections. Lancet [Internet]. (2018); 392:1341–1348. Available online at: https://linkinghub.elsevier.com/retrieve/pii/S0140673618319287 30322584 10.1016/S0140-6736(18)31928-7

[7] Villar J Carroli G Zavaleta N Donner A Wojdyla D Faundes A. Maternal and neonatal individual risks and benefits associated with caesarean delivery: multicentre prospective study. BMJ [Internet]. (2007) 335:1025. doi: 10.1136/bmj.39363.706956.55 17977819 PMC2078636

[8] Behague DP. Consumer demand for caesarean sections in Brazil: informed decision making, patient choice, or social inequality? A population based birth cohort study linking ethnographic and epidemiological methods. BMJ [Internet]. (2002) 324:942–2. doi: 10.1136/bmj.324.7343.942 11964338 PMC102326

[9] Eslambolchi L Mosadeghrad AM Taheri S Afshari M. Taxonomy of effective strategies to reduce unnecessary caesarean sections: a systematic review. East Mediterr Health J. (2021) 27:826–34. doi: 10.26719/emhj.21.046 34486719

[10] American College of Obstetricians and Gynecologists. Quality-improvement strategies for safe reduction of primary cesarean birth. Committee Statement (2025). Available online at: https://www.acog.org/clinical/clinical-guidance/committee-statement/articles/2025/04/quality-improvement-strategies-for-safe-reduction-of-primary-cesarean-birth 10.1097/AOG.0000000000005888 40245424

[11] Laurita Longo V Odjidja EN Beia TK Neri M Kielmann K Gittardi I. “An unnecessary cut?” multilevel analysis of health system drivers of caesarean section rates in Italy: a systematic review. BMC Pregnancy Childbirth. (2020) 20:770. doi: 10.1186/s12884-020-03462-1 33302920 PMC7731545

[12] Molina G Weiser TG Lipsitz SR Esquivel MM Uribe-Leitz T Azad T. Relationship between cesarean delivery rate and maternal and neonatal mortality. JAMA [Internet]. (2015) 314:2263. doi: 10.1001/jama.2015.15553 26624825

[13] Betrán AP Ye J Moller A-B Zhang J Gülmezoglu AM Torloni MR. The increasing trend in caesarean section rates: global, regional and national estimates: 1990-2014. PLoS One [Internet]. (2016) 11:e0148343. doi: 10.1371/journal.pone.0148343 26849801 PMC4743929

[14] Souza J Gülmezoglu A Lumbiganon P Laopaiboon M Carroli G Fawole B Caesarean section without medical indications is associated with an increased risk of adverse short-term maternal outcomes: the 2004-2008 WHO Global Survey on Maternal and Perinatal Health. BMC Med [Internet]. (2010);8:71. doi: 10.1186/1741-7015-8-71 21067593 10.1186/1741-7015-8-71 PMC2993644

[15] American College of Obstetricians and Gynecologists (College), Society for Maternal-Fetal Medicine Caughey AB Cahill AG Guise JM Rouse DJ. Safe prevention of the primary cesarean delivery. Am J Obstet Gynecol. (2014). 210:179–193. doi: 10.1016/j.ajog.2014.01.026 24565430

[16] Gupta S Nagabhushana S Kabra N Mittal P Gupta N Arora R. Predictors of successful induction of labor: a prospective observational study. Eur J Obstet Gynecol Reprod Biol [Internet]. (2020) 252:255–62. doi: 10.1016/j.ejogrb.2020.06.042

[17] Selin L Wallin G Berg M. Factors associated with successful induction of labor: a population-based cohort study. Acta Obstet Gynecol Scand [Internet]. (2021) 100:678–86. doi: 10.1111/aogs.14447

[18] Karlsson O Ladfors L Wennerholm UB Högberg U Cnattingius S Stephansson O. Induction of labor and risk of cesarean delivery in nulliparous women: a population-based cohort study. PLoS One [Internet]. (2022) 17:e0263685. doi: 10.1371/journal.pone.0263685 35213544 PMC8880764

[19] Gibbons L Belizán JM Lauer JA Betrán AP Merialdi M Althabe F. The global numbers and costs of additionally needed and unnecessary cesarean sections performed per year: overuse as a barrier to universal coverage. World Health Organization Report [internet]. (2010).

[20] ACOG. Committee opinion no. 745: mode of term singleton breech delivery. Obstet Gynecol. (2018) 132:e60–e63. doi: 10.1097/AOG.0000000000002755 30045211

[21] Vogel JP Betrán AP Vindevoghel N Souza JP Torloni MR Zhang J. Use of the Robson classification to assess caesarean section trends in 21 countries: a secondary analysis of two WHO multicountry surveys. Lancet Glob Health. (2015) 3:e260–70. doi: 10.1016/S2214-109X(15)70094-X 25866355

[22] Armstrong C. ACOG updates recommendations on vaginal birth after previous cesarean delivery. Am Fam Physician. (2011) 83:214–216.

[23] Betran A Torloni M Zhang J Gülmezoglu A. Section tWWGoC WHO statement on caesarean section rates. BJOG Int J Obstet Gynaecol. (2016) 123:667–70. doi: 10.1111/1471-0528.13526, PMID: 26681211 PMC5034743

[24] Campbell S Murphy M Keane DP Robson M. 413: classification of intrapartum cesarean delivery: a starting point for more detailed analysis. Am J Obstet Gynecol. (2017). 216:S413. doi: 10.1016/j.ajog.2016.11.671

[25] Ciriello E Locatelli A Incerti M Ghidini A Andreani M Plevani C. Comparative analysis of cesarean delivery rates over a 10-year period in a single institution using 10-class classification. J Matern Fetal Neonatal Med. (2012) 25:2717–20. doi: 10.3109/14767058.2012.712567, PMID: 22827562

[26] European Board and College Of Obstetrics And Gynaecology Ebcog. Ebcog position statement on caesarean section in Europe. Eur J Obstet Gynecol Reprod Biol. (2017). 219:129–132. doi: 10.1016/j.ejogrb.2017.04.018 28890279

[27] FIGO Statement. Best practice advice on the 10-group classification system for cesarean deliveries. Int J Gynaecol Obstet. (2016). 135:232–233. doi: 10.1016/j.ijgo.2016.08.001 27609739

[28] Le Ray C Blondel B Prunet C Khireddine I Deneux-Tharaux C Goffinet F. Stabilising the caesarean rate: which target population? BJOG Int J Obstet Gynaecol. (2015) 122:690–9. doi: 10.1111/1471-0528.13199, PMID: 25412695

[29] Pyykönen A Gissler M Løkkegaard E Bergholt T Rasmussen SC Smárason A. Cesarean section trends in the Nordic countries – a comparative analysis with the Robson classification. Acta Obstet Gynecol Scand. (2017) 96:607–16. doi: 10.1111/aogs.13108, PMID: 28176334

[30] Robson M. A global reference for CS at health facilities? Yes, but there is work to do. BJOG. (2016) 123:437. doi: 10.1111/1471-0528.13619, PMID: 26412795

[31] Robson MS. The 10-group classification system-a new way of thinking. Am J Obstet Gynecol. (2018) 219:1–4. doi: 10.1016/j.ajog.2018.05.026, PMID: 29941276

[32] Robson M. The ten Group classification system (TGCS) – a common starting point for more detailed analysis. BJOG. (2015) 122:701. doi: 10.1111/1471-0528.13267, PMID: 25600521

[33] Robson M Murphy M Byrne F. Quality assurance: the 10-group classification system (Robson classification), induction of labor, and cesarean delivery. Int J Gynaecol Obstet. (2015) 131:S23–7. doi: 10.1016/j.ijgo.2015.04.026, PMID: 26433499

[34] Robson MS. Classification of caesarean sections. Fetal Matern Med Rev. (2001). 12:23–39.

[35] World Health Organization. Robson classification: Implementation manual. Licence: CCBY-NC-SA3.0IGO. Geneva: World Health Organization (2017).

[36] World Health Organization. WHO statement on caesarean section rates (WHO/RHR/15.02). Geneva: World Health Organization (2015).

[37] Boerma T Ronsmans C Melesse DY Barros AJ Barros FC Juan L. Global epidemiology of use of and disparities in caesarean sections. Lancet. (2018) 392:1341–8. doi: 10.1016/S0140-6736(18)31928-7, PMID: 30322584

[38] Lafitte AS Dolley P Le Coutour X Benoist G Prime L Thibon P. Rate of caesarean sections according to the Robson classification: analysis in a French perinatal network – interest and limitations of the French medico-administrative data (PMSI). J Gynecol Obstet Hum Reprod. (2018) 47:39–44. doi: 10.1016/j.jogoh.2017.11.012, PMID: 29208502

[39] Sandall J Tribe RM Avery L Mola G Visser GH Homer CS. Short-term and long-term effects of caesarean section on the health of women and children. Lancet. (2018) 392:1349–57. doi: 10.1016/S0140-6736(18)31930-5, PMID: 30322585

